# Optical van-der-Waals forces in molecules: from electronic Bethe-Salpeter calculations to the many-body dispersion model

**DOI:** 10.1038/s41467-022-28461-y

**Published:** 2022-02-10

**Authors:** Alberto Ambrosetti, Paolo Umari, Pier Luigi Silvestrelli, Joshua Elliott, Alexandre Tkatchenko

**Affiliations:** 1 Dipartimento di Fisica e Astronomia, Università degli Studi di Padova, 35131 Padova, Italy; 2 School of Chemical Engineering and Analytical Science, University of Manchester, Manchester, UK; 3 Department of Physics and Materials Science, University of Luxembourg, L-1511 Luxembourg City, Luxembourg

**Keywords:** Chemical physics, Biological physics, Method development, Computational chemistry

## Abstract

Molecular forces induced by optical excitations are connected to a wide range of phenomena, from chemical bond dissociation to intricate biological processes that underpin vision. Commonly, the description of optical excitations requires the solution of computationally demanding electronic Bethe-Salpeter equation (BSE). However, when studying non-covalent interactions in large-scale systems, more efficient methods are desirable. Here we introduce an effective approach based on coupled quantum Drude oscillators (cQDO) as represented by the many-body dispersion model. We find that the cQDO Hamiltonian yields semi-quantitative agreement with BSE calculations and that both attractive and repulsive optical van der Waals (vdW) forces can be induced by light. These optical-vdW interactions dominate over vdW dispersion in the long-distance regime, showing a complexity that grows with system size. Evidence of highly non-local forces in the human formaldehyde dehydrogenase 1MC5 protein suggests the ability to selectively activate collective molecular vibrations by photoabsorption, in agreement with recent experiments.

## Abstract (web summary)

The authors devise an efficient quantum approach to address the van der Waals interactions due to photoexcitations by approximating the Bethe-Salpeter equation. Both attractive/repulsive forces can arise, that could couple to collective protein dynamics.

## Introduction

The optical excitation of molecules and materials is intimately connected to their mechanical response. Photoinduced electron-charge rearrangement—typically happening on the femtosecond scale—can be followed by conformational changes^1,2^, dissociation^3^, or chemical-bond strengthening^4^. Ensuing structural deformations have been identified as a source of efficiency loss in dye-sensitized solar cells^5^, and play a pivotal role in metal-to-ligand charge transfer^6^. Analogous mechanisms are equally relevant for biological processes. For instance, visible light conversion into electrical signals is triggered in retinal tissues by conformational changes of photopigments upon photoabsorption with single-photon sensitivity^7^. Moreover, steady emission and absorption of biophotons observed in brain cells suggest that light may provide a supplementary mechanism for information exchange^8^, in addition to electro-chemical signals. The relevance of opto-mechanical mechanisms is further highlighted by evidence of photoinduced vibrations^9^ in proteins, with occurrence of phonon condensation^10^.

While photoinduced response is predominantly identified with chemical-bond distortions, recent experiments^11^ provided direct evidence of unconventional van der Waals-like (vdW) interactions between Rydberg atoms at a Förster resonance. These interactions differ from conventional London’s dispersion (scaling as *R*^−6^), in that they exhibit a radically slower decay (*R*^−3^ scaling). Close analogy with photoexcited systems, where analogous interaction can arise, suggests experimental ability to induce and control molecular forces by optical excitation, targeting cold-atoms, self-assembly processes, and biological mechanisms. For instance, the existence of slowly-decaying vdW interactions (hereafter called optical) was predicted between two excited atoms^12^ also in the presence of perturbed interactions^13^, and between excited atoms and dispersive dielectric surfaces^14^. Also, external tuning of vdW forces between colloidal particles^15^ and between polar molecules^16^ was accomplished by applied light fields. We remark, however, that the optical vdW forces arising in complex systems, including molecules, proteins, or nanomaterials remain presently unexplored due to the difficulty in treating many-body effects.

A pioneering approach based on the GW-BSE (Bethe–Salpeter equation) framework was proposed by Ismail-Beigi and Louie^17^ to compute atomic forces in optically-excited molecules. While this framework can be applied to arbitrary systems, it is associated with a very high computational cost compared to pure GW-BSE energy calculations, due to the inclusion of Kohn-Sham (KS) orbital derivatives. While covalently-bonded systems require evaluation of wavefunction derivatives, in the case of well-separated molecules suitable approximations can be introduced. Under these circumstances, electron-hole excitations can be confined on single molecules, while long-range intermolecular correlations due to the Coulomb coupling between well-separated charge excitations must be treated explicitly.

## Results

Starting from BSE, here we introduce an effective theory based on coupled quantum Drude oscillators (cQDO) represented by the many-body dispersion^18,19^ (MBD) model. This approach enables extensive analysis of optical-vdW forces in large-scale photoexcited systems by inclusion of many-body effects. Our assessment of atomic hydrogen dimer, molecules, and 1D nanostructures illustrates the ability to externally switch between attractive and repulsive optical-vdW interactions, and demonstrates the rapidly-growing complexity of optical vdW forces with respect to the system size. Moreover, analysis of the human 1MC5 protein suggests that collective conformational changes and vibrations can arise upon optical excitation.

The BSE approach^20,21^, makes use of a Hamiltonian-like operator (see Supplementary Note 1 and ref. ^22^) ${\hat{H}}^{{{{{{{{\rm{eh}}}}}}}}}$ to account for neutral (electron-hole) excitations. Atomic units are adopted hereafter along with the Tamm-Dancoff approximation. In the case of (optically most relevant) spin-singlet excitations, the BSE operator can be expressed as

1 $${\hat{H}}^{{{{{{{{\rm{eh}}}}}}}}}=\hat{D}+2{\hat{K}}^{{{{{{{{\rm{x}}}}}}}}}+{\hat{K}}^{{{{{{{{\rm{d}}}}}}}}}\,.$$

Here, $\hat{D}$ is a diagonal term that accounts for single electron-hole energy transitions. ${\hat{K}}^{{{{{{{{\rm{x}}}}}}}}}$ is conventionally indicated as the BSE electron-hole exchange term (see Supplementary Note 1), and provides a coupling between different occupied-virtual electronic transitions. Finally, ${\hat{K}}^{d}$ is the so-called BSE direct term, that accounts for the interaction between overlapping particle-hole pairs. Intermolecular direct terms ${\hat{K}}^{{{{{{{{\rm{d}}}}}}}}}$ rapidly decay at long distance, due to a vanishing orbital overlap. Hence, when two molecules are far away from each other, only the longer-ranged intermolecular exchange ${\hat{K}}^{{{{{{{{\rm{x}}}}}}}}}$ matters.

We restrict now for simplicity to two identical molecules at separation distance *R* (taken along the $\hat{z}$ direction for reference). Extension to arbitrary geometries is straightforward. Under these assumptions, the total BSE Hamiltonian-like operator ${\hat{H}}^{{{{{{{{\rm{eh}}}}}}}},{{{{{{{\rm{T}}}}}}}}}$ can be conveniently decomposed into block matrices as follows:

2 $${\hat{H}}^{{{{{{{{\rm{eh}}}}}}}},{{{{{{{\rm{T}}}}}}}}}=\left(\begin{array}{cc}{\hat{H}}_{1}^{{{{{{{{\rm{eh}}}}}}}}}&{\hat{K}}_{12}^{{{{{{{{\rm{x}}}}}}}}}\\ {\hat{K}}_{12}^{{{{{{{{\rm{x}}}}}}}}\,{{{\dagger}}} }&{\hat{H}}_{2}^{{{{{{{{\rm{eh}}}}}}}}}\\ \end{array}\right)\,.$$

Diagonal blocks contain BSE operators relative to the isolated molecules (labeled as 1,2). Off-diagonal exchange blocks determine the Coulomb coupling between charge rearrangements arising in the two molecules upon optical excitation. ${\hat{K}}_{12}^{{{{{{{{\rm{x}}}}}}}}}$ will essentially coincide with its dipolar counterpart in the long range, producing either alignment or antialignment between molecular dipole excitations. As a consequence, single-molecule excitation frequencies (degenerate in the absence of intermolecular coupling) undergo a splitting which depends on *R*.

An implementation of BSE based on dipole approximation and on the BSE-Simple code^22^ was developed in the context of this work (see Supplementary Note 1 and ref. ^22^ for more details). The screened interaction is computed at the GW level^23–25^, and a scissor^26^ for KS energy eigenvalues (namely a rigid shift of the KS energies relative to virtual orbitals only) is introduced accordingly. The present density-functional theory and GW-BSE calculations are performed via the Quantum Espresso^27^ code, adopting the Perdew–Burke–Ernzerhof (PBE) functional^28^ for the evaluation of single-particle orbitals.

**Fig. 1:**
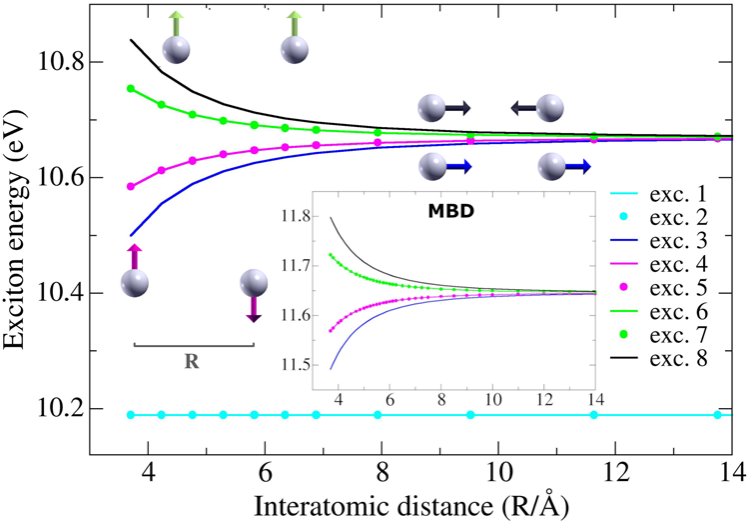
Exciton energies in the hydrogen dimer. BSE excitation energies (i.e., energy required by the system to excite a given optical mode) are reported as a function of the interatomic separation *R* (MBD excitation energies are shown as inset). The first two BSE excitation modes (cyan) correspond to 1*s* → 2*s* transitions and undergo no dipolar splitting due to their spherical symmetry. BSE excitations 3–8 instead correspond to 1*s* → 2*p* transitions (degenerate only in the limit of vanishing interatomic coupling), and exhibit growing splitting at short interatomic distance. Optical vdW forces for a given excitation mode correspond to the inverse of the exciton-energy gradient. Gray balls schematically indicate atoms, while arrows (whose colors replicate the corresponding energy curves) give intuitive description of the correlation arising between dipolar displacements in the two H atoms. In excitons 3 and 8 (blue and black, respectively), atomic dipole modes are oriented along the direction joining the two atoms. Parallel dipoles (exc. 3) imply attractive coupling, while repulsion is found when the two dipoles are antiparallel, in analogy to the electrostatic case. Similar mechanisms arise in excitons 4–7, where dipole moments are instead oriented orthogonally to the H–H direction.

Figure 1 reports the low-lying excitation spectrum of two H atoms as a function of interatomic distance *R*. Given the availability of analytical electronic levels for atomic H, the single-atom BSE Hamiltonian is identified with $\hat{D}$ only, upon introduction of a suitable scissor^26^ for KS eigenvalues, to reproduce the correct 1*s* → 2*s* transition energy. The two lowest-energy BSE excitations (corresponding to 1*s* → 2*s* transitions) exhibit no detectable splitting. However, excitation levels corresponding to 1*s* → 2*p* transitions are clearly split by the intermolecular interaction. BSE essentially provides the energy difference between ground state and optically excited levels. Hence, a splitting or variation of the exciton energy (${E}_{{{{{{{{\rm{exc}}}}}}}}}^{i}$ for the *i*-th mode) with respect to *R* implies the emergence of non-vanishing optical vdW forces. These forces can be defined as the inverse of the exciton-energy gradient with respect to the geometrical coordinates

3 $${{{{{{{{\bf{F}}}}}}}}}_{{{{{{{{\rm{opt}}}}}}}}-{{{{{{{\rm{vdW}}}}}}}}}^{i}=-{{{{{{{\boldsymbol{\nabla }}}}}}}}{E}_{{{{{{{{\rm{exc}}}}}}}}}^{i}\,,$$

and coexist with other forces (such as dispersion, covalent, or electrostatic interactions), already present in the ground state. We also note that optical excitations can interplay with short-ranged interactions, but such effects will not be considered in our work. Both attractive (lower branch, monotonically increasing with *R*) and repulsive (upper branch, decreasing with *R*) interactions are obtained by selection of the excitation symmetry (dipole alignment/antialignment). Taking the dipole limit of ${\hat{K}}_{12}^{{{{{{{{\rm{x}}}}}}}}}$, one finds that the coupling between single-atom excitations depends on *R*, and on the dipole transition-matrix elements between occupied and virtual electronic states (see Supplementary Note 1). The change in angular momentum in 1*s* → 2*p* transitions implies finite dipole-matrix elements, so that the initially six-fold-degenerate 1*s* → 2*p* excitations in the two H atoms are split into four different levels (full degeneracy is only recovered in the limit of *R* → *∞*). Due to rotational symmetry, *x* and *y* dipole excitations remain degenerate and undergo identical attraction/repulsion, whereas *z* dipole transitions involve larger splitting. Vanishing dipole elements characterize instead 1*s* → 2*s* excitations (modes 1,2), due to the spherical symmetry of *s* orbitals, which explains the negligible splitting.

To further inspect the optical-vdW interaction between excited systems, we now adopt the effective many-body dispersion (MBD) model^18,29^, based on a first-principles^30^ parametrization. MBD maps the dipolar response of the atoms into a set of coupled quantum Drude oscillators (cQDO), according to the following Hamiltonian^18,19,29^:

4 $${H}^{{{{{{{{\rm{MBD}}}}}}}}}=\mathop{\sum }\limits_{i=1}^{N}\left(-\frac{{\bigtriangledown}_{{{{{{{{{\boldsymbol{\xi }}}}}}}}}_{i}}^{2}}{2}+\frac{{\omega }_{i}^{2}{{{{{{{{\boldsymbol{\xi }}}}}}}}}_{i}^{2}}{2}\right)+\mathop{\sum }\limits_{i\ne j}^{N}\mathop{\sum }\limits_{\beta ,\gamma =1}^{3}{\theta }_{{{ij}}}{\xi }_{i}^{\beta }{T}_{ij}^{\beta \gamma }{\xi }_{j}^{\gamma }\,,$$

with ${\theta }_{ij}={\omega }_{i}{\omega }_{j}\sqrt{{\alpha }_{i}^{0}{\alpha }_{j}^{0}}$. Here ${\xi }_{i}^{\beta }$ represents the *β* Cartesian component (*β* ∈ {*x*, *y*, *z*}) of the mass-weighted charge displacements^31^ from the *i*-th ion position **R**_*i*_. The tensor *T* introduces the inter-oscillator dipole coupling, and it is defined as ${T}_{ij}^{\gamma \delta }={\partial }_{{R}_{i}^{\gamma }}{\partial }_{{R}_{j}^{\delta }}v({R}_{ij})$, where *v*(*R*_*ij*_) is the Coulomb interaction between atoms *i* and *j* damped at short range due to Gaussian charge overlap^29^. The introduction of a suitable damping function for the Coulomb coupling ensures a range-separation^19,32^ of correlation effects, so that only long-range vdW terms are included in MBD, while short-range contributions such as deriving from covalent bonding are neglected. This approach allows straightforward application of MBD to overlapping atoms, in analogy to standard methods for vdW dispersion interactions^30^. Even in dense systems MBD is specifically designed to describe the long-range charge-fluctuation modes of relevance to vdW forces. Comparing Eqs. (2) and (4), we note that MBD accounts for dipolar exchange coupling between different atoms, while modeling atomic electron transitions through effective QDO excitations having angular momentum variation Δ*L* = 1. In fact, the MBD two-body coupling (last term in Eq. (4)) annihilates/constructs single excitations in pairs of QDO’s (*i* and *j*), thereby involving coupled transitions between occupied/virtual states in analogy with the BSE exchange term (see Supplementary Note 1).

Diagonalization of *H*^MBD^ for *N* atoms yields a set of collective oscillator modes with frequencies ${\bar{\omega }}_{i}$ (*i* = 1, . . , 3*N*). By fixing *N* = 2 and taking identical static polarizabilities *α*^0^ and characteristic frequencies *ω* (i.e., identical atoms), the following renormalized oscillator eigenmodes are found (we report both frequency and dipole-fluctuation geometry):

5 $$\begin{array}{lll}{\bar{\omega }}_{1,2}&=&\omega \sqrt{1\pm {\tilde{R}}^{-3}}\qquad \frac{{\xi }_{1}^{x}\pm {\xi }_{2}^{x}}{\sqrt{2}}\,,\\ {\bar{\omega }}_{3,4}&=&\omega \sqrt{1\pm {\tilde{R}}^{-3}}\qquad \frac{{\xi }_{1}^{y}\pm {\xi }_{2}^{y}}{\sqrt{2}}\,,\\ {\bar{\omega }}_{5,6}&=&\omega \sqrt{1\pm 2{\tilde{R}}^{-3}}\qquad \frac{{\xi }_{1}^{z}\,\mp\, {\xi }_{2}^{z}}{\sqrt{2}}\,,\end{array}$$

where ${\tilde{R}}^{-3}={\alpha }^{0}{R}^{-3}$. By definition, the MBD ground-state energy is ${E}_{0}^{{{{{{{{\rm{MBD}}}}}}}}}=\mathop{\sum }\nolimits_{i = 1}^{3N}{\bar{\omega }}_{i}/2$, which automatically accounts for London dispersion, and scales here as *R*^−6^. The scaling can be readily obtained by Taylor expansion of the above frequencies: first-order terms in ${\tilde{R}}^{-3}$ are canceled out after summation, and only second-order perturbative terms (proportional to ${\tilde{R}}^{-6}$) survive (constant terms being irrelevant). MBD excited states (also called excitons hereafter for simplicity) can be obtained by exciting one (or more) collective cQDO modes, thereby increasing the ground-state energy *E*_0_ by an integer multiple of the renormalized oscillator frequencies ${\bar{\omega }}_{i}$. As independently observed by other authors^12,33^, and in agreement with our BSE calculations, we find that the resulting interatomic interaction scales as *R*^−3^. In fact, while dispersion emerges as a second-order perturbative term, the optical vdW interaction arising between two atoms is a first-order effect: no cancellation occurs when only a single mode is excited. Moreover, inspection of modal geometries confirms that attraction or repulsion arises from the interaction between aligned or antialigned dipole excitations (see Fig. 1).

Fair agreement between MBD and BSE 1*s* → 2*p* transitions exists for the atomic H dimer. Also, from Eq. (5) we observe that *z* dipole-oscillations imply larger splitting due to the anisotropy of the *T* tensor, which clearly rationalizes the above BSE results. By construction, MBD neglects 1*s* → 2*s* transitions, since dipole excitation of QDOs implies finite variation in the angular momentum. This explains the absence of flat levels in the MBD spectrum of Fig. 1.

**Fig. 2:**
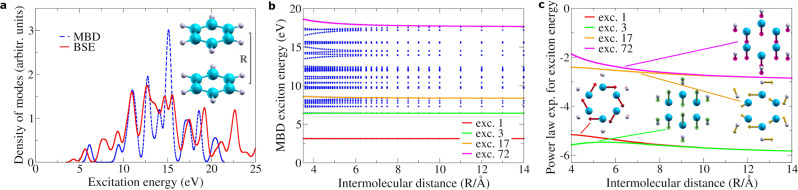
Analysis of exciton spectra and optical vdW interactions in the benzene dimer. **a** Comparison between MBD and BSE excitation spectra for benzene. The MBD spectrum is rigidly shifted by +3 eV. A Gaussian broadening of 0.3 eV was introduced in MBD and BSE calculations (and adopted hereafter). **b** MBD exciton energies for the benzene dimer as a function of the intermolecular distance *R* (in analogy to Fig. 1). Select exciton modes, relevant to our analysis (1,3,17,72) are highlighted with colored lines to facilitate visualization. The lowest-energy excitation with visible splitting (exc. 17) is at ~8.38 eV and involves sizeable dipole transition. Lower-energy modes (such as exc. 1,3) undergo smaller splitting due to more complex charge rearrangement. **c** Power-law exponents for select excitation modes (highlighted in **b**), computed as the slope of the MBD exciton energy vs. *R* in logarithmic scale^35^. The geometry of these modes is displayed in schematic form, with arrows indicating atomic dipoles. Modes 17 and 72 have finite (overall) dipole moments and exhibit an asymptotic behavior of *R*^−3^, whereas modes 1 and 3 have negligible dipole moment, and show ~*R*^−6^ power law.

While the effective MBD model unavoidably introduces spectral simplifications (due to coarse graining over the electronic charge), it represents a computationally efficient tool for the analysis and physical interpretation of non-covalent optical-vdW forces between large photoexcited systems (up to ~10^4^ atoms). We thus extend MBD predictions beyond diatomic systems, considering the benzene dimer (see Fig. 2). As a first step, we compare the full MBD and BSE excitation spectra (see Fig. 2a) of benzene. By introducing a +3 eV rigid shift on the MBD spectrum (a commonly adopted procedure in spectral analysis^34^, that essentially corresponds to a renormalization of the energy-gap between highest-occupied and lowest-unoccupied molecular orbitals), we observe semi-quantitative agreement with BSE. We stress that the qualitative agreement between BSE and MBD spectra appears to be a recurrent feature: in fact, further comparison over a set of six different molecules (namely, methane, ammonia, e-butadiene, naphtalene, pyrazine, and all-e-octatetraene—see Supplementary Note 2) consistently leads to analogous conclusions. We thus argue that structural symmetries and exchange coupling (both present in MBD by construction) play a major role in determining collective charge displacements. Remaining discrepancies, on the other hand, are ascribed to the absence of delocalized charge transfer and covalent bonding within MBD.

In Fig. 2b, we observe a number of weakly- or negligibly-split modes at low energy, while the first significant splitting in the MBD spectrum is found around 8 eV. In order to interpret weakly-split levels we remark that collective MBD excitations can correspond to complex internal arrangements of the atomic dipoles, that can result in either negligible or significant macroscopic molecular dipoles. Depending on the overall arrangement of the single atomic dipoles, different power-law scalings are found for the corresponding exciton splitting (see SI). We also recall that the above BSE approach for optical vdW interactions can only capture the splitting of those excitons associated to macroscopic dipole moments within the simulation cell. This approximation becomes increasingly crude in complex systems, where charge oscillations often arrange in complex patterns with negligible overall dipole moment.

**Fig. 3:**
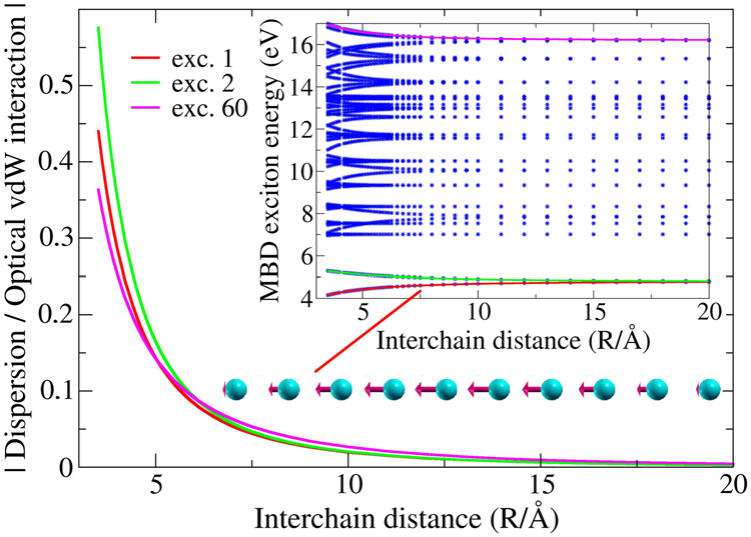
Ratio between dispersion and optical-vdW interchain interaction energies for two parallel carbyne-like chains. The ratio (absolute value) between the interaction energy due to optical vdW interactions and dispersion is reported for three largely split MBD exciton modes (1,2,60), arising in parallel chains with 10 carbon atoms each (nearest-neighbor separation of 1.4 Å). Optical vdW interactions largely dominate over dispersion already at a few Å scale. The optical vdW interaction energy is defined as the difference between a given exciton energy computed at finite *R* and in the *R* → *∞* limit. Inset: MBD exciton energies (blue dots), as a function of the interchain distance *R*. The analyzed excitons (1,2,60) are highlighted by colored lines, and the geometry of the plasmon-like mode 1 (over a single chain only, being the two chains identical) is illustrated. Higher-frequency longitudinal excitations will display one or more nodes (inversion points) along the structure. Identical modes in the two chains either correlate or anticorrelate due to the Coulomb coupling, leading to the observed exciton splitting.

To address optically-induced interactions between extended nanostructures we now analyse parallel one-dimensional carbyne-like chains, containing 10 atoms each. From Fig. 3, we observe a large splitting in the first excitation mode. Geometrically, this mode corresponds to a longitudinal charge displacement which coherently propagates atom by atom through the entire chain^35^, resulting in a macroscopic dipole excitation. In analogy to the H dimer, identical modes in the two chains can correlate or anticorrelate due to the Coulomb coupling, producing exciton splittings that vary as a function of *R*. Higher-energy modes exhibit variable features, and tend to form a continuum, which becomes more evident in longer chains (see Supplementary Note 3). We also underline that, in the limit of infinite chains, the low-lying excitation spectrum qualitatively agrees (except for a small gap due to the finite gap of QDO’s) with the analytic spectrum of 1D metals^35,36^, providing further validation to the MBD approach.

So far, we only focused on excitation energies. However, London dispersion interactions (naturally inbuilt in the MBD ground-state^18^ energy ${E}_{0}^{{{{{{{{\rm{MBD}}}}}}}}}$) also contribute to the total interfragment interaction. Scaling arguments already suggest that (~*R*^−3^) exciton splitting terms will dominate over (~*R*^−6^) dispersion contributions at the long range, as numerically confirmed for both atomic dimers and benzene. In the case of large nanostructures, however, both dispersion^35,36^ and excitation splittings may show non-trivial scaling due to the strong coherence and macroscopic scale of the many-body polarization modes. In Fig. 3, the interchain interaction energy due to London dispersion is compared to the optical vdW interaction in order to reveal the total-energy dependence on *R*. In the 10-atom chain considered here, the dispersion interaction is already negligible at the 0.5 nm scale. However, in a 100-atom chain (see Supplementary Note 3) dispersion effects are much larger, and tend to vanish only at *R* ~ 1.5 nm. We accordingly expect major interplay between dispersion and exciton mode splitting in large nanostructures or biomolecules.

**Fig. 4:**
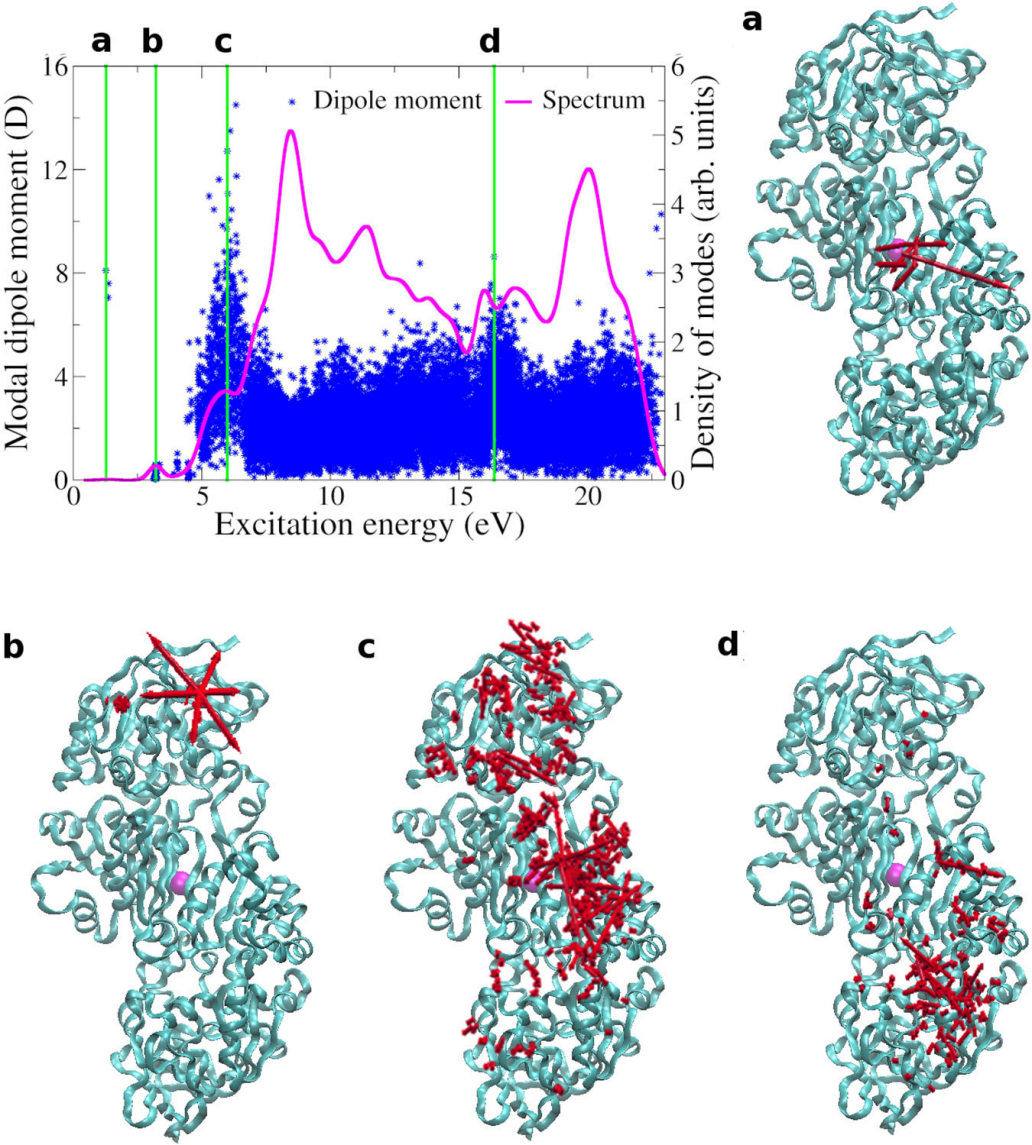
Analysis of MBD exciton modes for the human protein 1MC5. The MBD excitation spectrum is reported together with the overall dipole moment (absolute value) characterzing each excitation mode. Non-covalent optical-vdW forces (illustrated as red arrows) are reported for select excitation modes (**a**–**d**), indicated in the spectrum by green vertical lines. The position of the potassium site close to the protein center is indicated with a magenta sphere. Relatively localized dynamics are associated to low-frequency excitations, whereas remarkably collective forces are found at higher frequencies.

Given the relevance of optical excitations in biological systems, we finally consider the 1MC5 protein (human glutathione-dependent formaldehyde dehydrogenase), which is involved in the metabolism of glutathione adducts. The protein is composed of 6069 atoms, and due to the large size and soft structure, internal optical-vdW forces arising upon photoexcitation can play a major role. We recall that the inclusion of a short-range damping for the dipole interaction in MBD allows to extend our analysis to relatively compact structures^18^, beyond the well-separated fragments configurations. In practice, here we extend the computation of optical vdW interactions to intrafragment terms, in analogy to the dispersion energy. In the case of well-separated fragments (such as two atoms or molecules) one defines the intermolecular dispersion interaction energy as the energy difference between the two molecules at finite separation *R* and at *R* → *∞*. Nonetheless, dispersion interactions also arise within a single molecule (or protein in this case), basically coexisting with covalent or electrostatic interactions. Intramolecular dispersion forces become extremely relevant in proteins due to their large extension and structural flexibility, and were shown to influence the balance between folded and unfolded conformations^37^. The intramolecular dispersion energy is accordingly defined as the vdW energy difference between the considered configuration and the uncoupled (or non-interacting) atoms. The remaining challenge is how to separate vdW dispersion from covalent-bond energies. The standard way to proceed^19,30^ is to cut the vdW energy contribution at short distances by introducing a suitable damping function. With this procedure, cohesive energies due to covalent bonds (derived for instance from semi-local density-functional theory) can be straightforwardly added to the vdW contribution, implying an effective range-separation^32^ of the correlation energy. Here, an analogous strategy is followed for optical vdW forces: as previously mentioned, MBD relies on a damping function which cuts the interatomic coupling at the short range, thereby separating optical vdW interactions from shorter-ranged contributions (which can coexist, but are beyond the scope of this work). Since optical vdW forces are directly computed from the gradient of the MBD exciton energy (Eq. (3)), manual separation into fragments or comparison with the *R* → *∞* limit is not necessary in practice. We remark that, while MBD is a coarse-grained model that does not account for covalent bonds, it can reproduce the many-body polarizability of molecules and bulk materials^38^ within ~5% accuracy, since it captures the collective charge oscillations relevant to vdW forces. We also note that short- and long-ranged correlation terms are associated to different energy scales, so that the coupling between the two is expected to be small. Moreover, at the optimal geometry the system is at equilibrium when no excitation is present, so that only excitation-induced forces can play a role in inducing protein distortion. In Fig. 4 we report the MBD excitation spectrum, and the forces arising in the presence of select excitations. Surprisingly, lowest frequencies consistently correspond to relatively localized forces, which typically involve finite stress in proximity of the potassium atom. This suggests a relevance of the potassium site as a possible mean for activation of optically-induced conformational changes. At slightly higher frequency, on the other hand, correlated distortions in different areas of the molecules are noticeable. Finally, beyond ~5 eV higher collectivity is consistently observed, suggesting highly non-local geometry rearrangements. These rearrangements can involve both internal atoms, or interfacial areas, and are thus likely to involve bending or interaction with external molecules. Depending on the excitation frequency range, different functionalities may thus be selected. We also note that short-ranged forces (neglected in this work) may equally arise upon optical excitations. However, these are expected to couple to localized geometrical rearrangements rather than to collective phonon modes.

## Discussion

Optical vdW interactions arise due to the correlation between dipole excitations in separated molecular fragments. Since the correlation inbuilt in the wavefunction is independent from the interfragment distance *R* (as it only depends on collective coordinates—see Eqs. (5)), the optical vdW forces emerge as a first-order contribution in the dipole-dipole coupling. In other words, while the expectation value of single-atom dipole-operators *ξ*_*i*_ over the MBD excitonic wavefunction vanishes, the correlation between fragments *i* and *j* produces finite expectation values for the product-operators *ξ*_*i*_*ξ*_*j*_. This implies that optical-vdW interactions should scale as ~*R*^−3^ in the presence of non-vanishing dipolar excitations (see Eqs. (5)). This can be understood intuitively, by analogy with the electrostatic coupling between permanent and induced dipoles.

Depending on the system, alternative excitations may also arise, which can induce optical-vdW interactions with shorter power-law decay (~*R*^−6^), as visible from Fig. 3. These MBD exciton modes are associated in benzene to vanishing overall dipole moments. In fact, while single atoms still undergo finite charge displacement, symmetric arrangements of atomic dipoles can eventually lead to overall cancellation (see Supplementary Note 4 for more detailed discussion). The splitting of wave-like excitation modes arising in the linear carbyne chains (see Fig. 3) can also exhibit *R*^−5^ scaling when these modes are antisymmetric with respect to the chain midpoint. Here, antisymmetry implies vanishing overall dipoles and quadrupole-quadrupole coupling at first order (see Supplementary Note 4).

In the context of linear carbyne-like chains, the interchain dispersion energy displays monotonic growth with respect to the system size, due to the increasing number of excitation modes involved. Conversely, the splitting of the (single) lowest-energy MBD excitation mode tends to decay at increasing chain length, essentially due to larger average interatomic separation. The ratio between dispersion and optical vdW interactions accordingly tends to decrease in the longer chains (see Supplementary Note 3 for comparison). Nonetheless, due to the essentially slower scaling of optical vdW interactions, excitations with finite dipole moment unavoidably provide the leading interaction term at the long range. We also note that the range where optical vdW interactions dominate over dispersion increases only by a factor ~3 passing from 10-atom to 100-atom chains.

In addition to single excitons, multiple excitations can be introduced, possibly leading to enhancement phenomena. On the other hand, when both attractive and repulsive modes are excited, effective enhancement of the many-body dispersion interaction is found. In fact, we recall that dispersion forces are ultimately due to the ground-state occupation of all available modes. Clearly, the rich variety of exciton modes discussed so far implicitly suggests viable pathways for the detailed external manipulation of vdW interactions; depending on the specific application, optical vdW forces can be potentially turned from attractive to repulsive, further altering both their strength and power-law decay.

We underline that optical vdW forces are expected to play a major role in the mechanical response of proteins and large biomolecules. In fact, due to the large size and high flexibility of these systems, long-ranged dispersion forces were found to crucially influence both geometry and stability^39^. Notably, experiments conducted by Nardecchia et al.^10^ revealed efficient energy transfer from optical excitations to collective vibrational modes. The underlying energy-conversion mechanism appears compatible with the highly non-local forces observed in Fig. 4 above ~5 eV: the non-locality of optical vdW forces, extending to large part of the structure, suggests effective coupling to collective vibrational modes, providing effective mechanisms for the experimentally-observed energy transfer. This observation is further corroborated by the cooperative coupling^40^ existing between many-body vdW interactions and ionic lattices, implying non-local force sensitivity^41^ to structural deformations. We also point out that the collective plasmon-like modes emerging in the 1MC5 protein could also be associated to the oscillation of finite dipole moments (see Fig. 4). For instance, mode (c) (see again Fig. 4) combines both non-local forces and non-vanishing overall dipole moment (computed as the vectorial sum of atomic dipoles), and analogous considerations hold for neighboring modes. While dipole moments associated to single modes are within the ~10 D scale (~13 D for mode (c)), the abundance of available modes could lead to constructive multimodal oscillation and amplification of intermolecular interactions. As a consequence, we speculate that plasmon-like charge oscillation modes could effectively participate in the ordered dynamics of living matter, whenever optical excitations take place. In this regard, we remark that photoluminescence is a well-known phenomenon in living tissues^42^, and biophoton exchange is believed to play a major role in neural information-processing and encoding^43^. We finally remark that finite temperatures play a negligible role in optical vdW forces, due to the relatively high energy cost of excitonic modes (see Supplementary Note 6).

In conclusion, we introduced an effective model for semi-quantitative analysis of vdW forces in optically excited nanostructures and extended systems. Optical excitations can be exploited to induce both attractive and repulsive long-range forces between well-separated molecules, depending on the geometrical symmetry of excitations. The complexity of these forces grows rapidly with system size, due to the intricate geometrical features of collective atomic forces. Photoinduced vdW forces dominate over vdW dispersion in the long-range distance regime. Notably, the analysis of the 1MC5 human protein reveals unexpected energetic separation between excitation modes inducing either highly collective or more localized stress in the system. The non-locality of emergent optical vdW forces suggest effective coupling to collective vibrational modes, and energy transfer between excitons and phonons, in agreement with the experimental evidence^10^ of phonon condensation upon optical light adsorption. This mechanism is also in a qualitative agreement with the experimental evidence^44^ of excitation-driven intramolecular dynamics of fluorescent proteins.

## Methods

The MBD method is discussed in refs. ^19,29,35^, where many-body vdW terms are addressed in detail for different systems. Dispersion interactions are due to the energy shift of collective charge-fluctuation modes in their ground state, caused by interatomic couplings. Analogously, optical vdW interactions, arise from the geometry-dependent energy shift of collective charge-fluctuation excitations.

## Supplementary information

Supplementary Information

## Data Availability

All relevant data presented in this paper are available from the corresponding author upon reasonable request.

## Code availability

The modified MBD code developed to extract exciton spectra is available, within the terms of the MIT licence. The MBD code is also accessible at http://www.fhi-berlin.mpg.de/tkatchen/MBD/.
