# Supplementary material for: Optical van-der-Waals forces in molecules: from electronic Bethe-Salpeter calculations to the many-body dispersion model
Source: Nat Commun. 2022 Feb 10;13:813. doi: 10.1038/s41467-022-28461-y (PMC8831584; doi:10.1038/s41467-022-28461-y)
Supplement: Supplementary file 1 — Supplementary Information [file 41467_2022_28461_MOESM1_ESM.pdf]

Supplementary Information
Optical van-der-Waals Forces in Molecules: From Electronic Bethe-Salpeter Calculations to the Many-Body Dispersion Model

A. Ambrosetti et al.

Supplementary Note 1

The BSE approach [1, 4], makes use of a Hamiltonian-like operator $\hat{H}^{\mathrm{eh}}$ to account for neutral (electron-hole) excitations. Atomic units are adopted hereafter to simplify the notation. Within the Tamm-Dancoff approximation the excitonic wave functions (eigenstates of $\hat{H}^{\mathrm{eh}}$) are written as $|\Theta\rangle = \sum_{vc} A_{vc}\hat{a}_v\hat{a}_c^\dagger|\phi_0\rangle$, where A_{vc} are weighting factors and $\hat{a}_i$, $\hat{a}_i^\dagger$ are the annihilation and creation operators for the i-th electronic state (whose wave function we will denote as ψ_i). ϕ_0 is the KS Slater determinant relative to the electronic ground state, and v,c indices run over valence and conduction states, respectively. In the case of (optically most relevant) spin-singlet excitations, the BSE operator can be expressed as

$$\hat{H}^{\mathrm{eh}} = \hat{D} + 2\hat{K}^{\mathrm{x}} + \hat{K}^{\mathrm{d}}\,. \tag{1}$$

Here, $\hat{D}$ is a diagonal term of the form $D_{vc,v'c'} = (\epsilon_c - \epsilon_v)\delta_{v,v'}\delta_{c,c'}$, ϵ_i being the i-th KS energy eigenvalue. $\hat{K}^{\mathrm{x}}$ is conventionally indicated as the BSE exchange term, and provides a coupling between different occupied-virtual electronic transitions in the system. Assuming that KS orbitals are real-valued, its components are defined as:

$$K^{\mathrm{x}}_{vc,v'c'} = \int \psi_v(\mathbf{r})\psi_c(\mathbf{r})v(\mathbf{r},\mathbf{r}')\psi_{v'}(\mathbf{r}')\psi_{c'}(\mathbf{r}')d\mathbf{r}d\mathbf{r}'\,. \tag{2}$$

Finally, the so-called BSE direct term is written as

$$K^{\mathrm{d}}_{vc,v'c'} = \int \psi_c(\mathbf{r})\psi_{c'}(\mathbf{r})w(\mathbf{r},\mathbf{r}',\omega)\psi_v(\mathbf{r}')\psi_{v'}(\mathbf{r}')d\mathbf{r}d\mathbf{r}'\,, \tag{3}$$

where $w(\mathbf{r},\mathbf{r}',\omega)$ is the screened Coulomb interaction.

Supplementary Note 2

We report in Supplementary Figure 1 a comparison between MBD and BSE excitation spectra for a set of six molecules, namely methane, ammonia, e-butadiene, naphtalene, pyrazine and

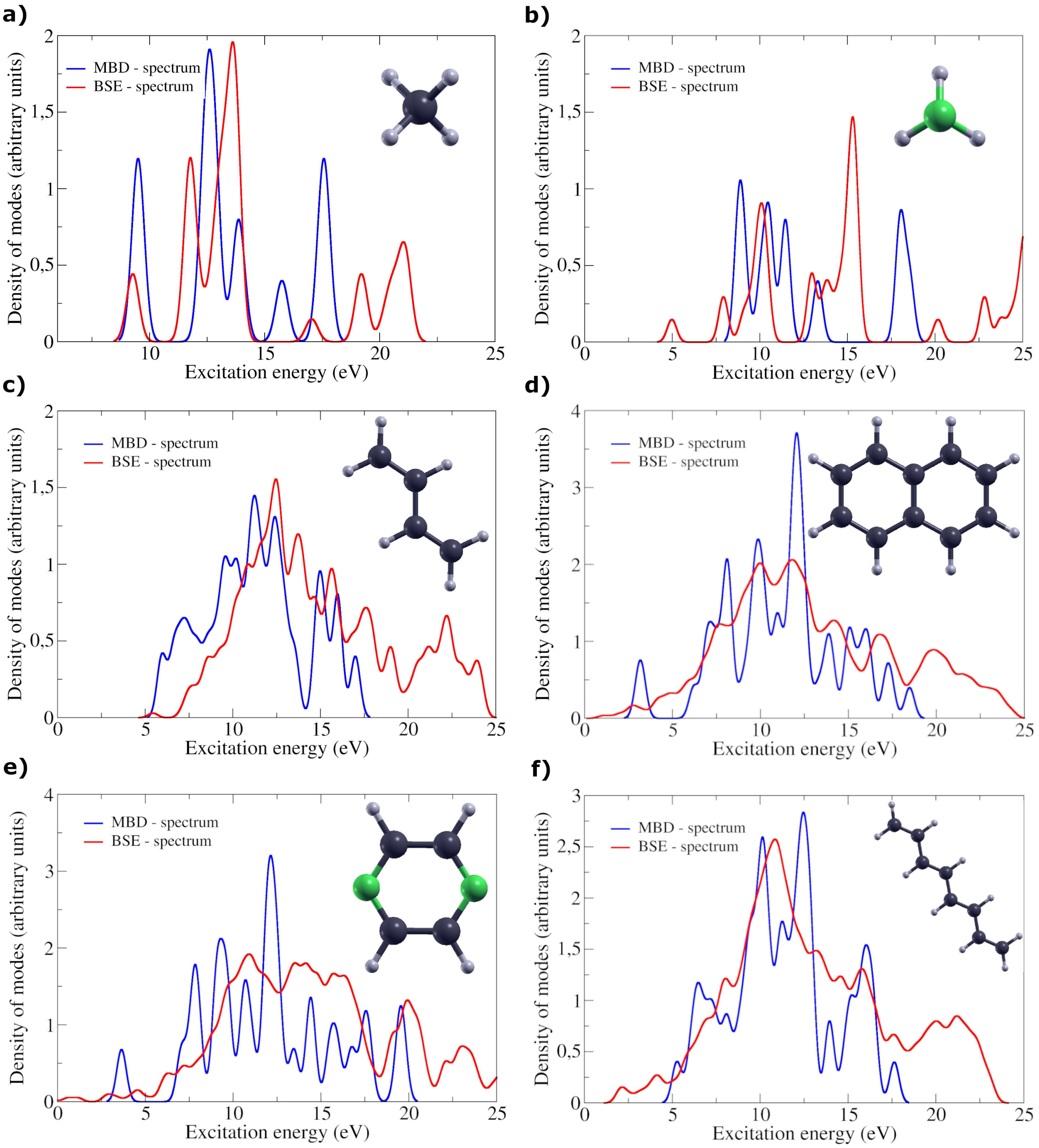

Supplementary Figure 1: Comparison between MBD and BSE excitation spectra for different molecules: **a)** methane, **b)** ammonia, **c)** e-butadiene, **d)** naphtalene, **e)** pyrazine and **f)** all-e-octatetraene. The methane MBD spectrum is rigidly shifted by +2 eV, while no shift was introduced for other molecules, in order to evidence the overall tendency towards red shifts in MBD spectra.

all-e-octatetraene. The chosen molecules cover a relatively broad spectrum of sizes, and configurations. However, generally fair qualitative agreement is consistently found between MBD and BSE results. Overall, MBD spectra tend to be redshifted and squeezed over smaller frequency intervals, although exhibiting analogous features to BSE. Although MBD relies on quasiparticle approximations and simplified atomic excitation spectra, spectral similarity is likely preserved by the relevant role of geometry and symmetries in determining collective charge-displacement modes. In fact, detailed geometrical features are naturally detected by the MBD method, due to the presence of interatomic couplings. On the other hand, we recall that MBD is a coarse-grained model that does not account for delocalized charge transfer, or short-range correlation terms. In principle, one may compare single-atom effective MBD frequencies to the relevant electronic transitions of the system, although a one-to-one correspondence is clearly not possible. We note that in the specific case of H, MBD naturally provides a reasonable exciton energy of ~ 11.5 eV, while pure PBE Kohn-Sham orbitals underestimate this energy gap by a factor of ~ 3. We stress in any case that any rigid shift of the MBD exciton spectrum has no influence on optical vdW forces, since these are obtained from energy derivatives.

Supplementary Note 3

While London dispersion (computed from the MBD model as well) is essentially negligible in small molecules, in the case of larger systems it can become relevant. In Supplementary Figure 2 we analyze a 1D chain composed of 100 C atoms. Here the interchain interaction energy due to London dispersion is larger than the exciton splitting up to the nm scale. At larger distance, though, exciton splitting prevails due to its slower power law decay. One can thus generally assume that London dispersion will acquire more relevance in larger structures, but photoinduced vdW forces will finally prevail in the long range limit.

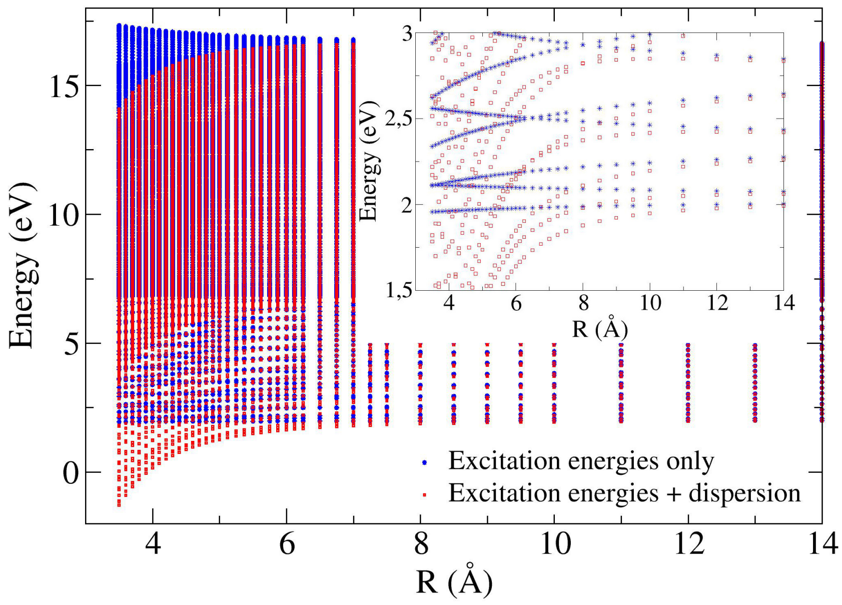

Supplementary Figure 2: MBD excitation energies (blue) for a 100 C chain. The nearest-neiighbor separation is fixed to 1.4 Å. London dispersion interchain binding energy is added to MBD excitation frequencies for comparison (red). Detail is provided in the inset.

Supplementary Note 4

In the presence of MBD exciton modes characterized by macroscopic dipole, interfragment optical vdW interactions scale as R^{-3} in the long-range limit. However, the situation substantially varies when the excitation symmetry implies vanishing overall dipole. For instance, MBD excitons 1 and 3 in benzene (See Supplementary Figure 2 -**c**) in the main paper) exhibit R^{-6} scaling. This is essentially due to a cancellation between first-order perturbative terms in Eq. 5) of the main paper, as a consequence of the vanishing total dipole moment. Second-order perturbative terms provide here the leading contribution to optical vdW interactions. On the other hand, in the case of linear carbyne-like C chains, the energy splitting of plasmon-like modes whose symmetry implies overall dipole cancellation can display R^{-5} scaling. The difference between benzene and linear chains resides in the overall symmetry of the mode, which determines different cancellation effects.

We analyze here in detail the case of carbyne-like chains. If the plasma-like excitation is longitudinal and antisymmetric (i.e. the wave has an odd number of nodes), then the total dipole is zero. The mode arising in a single chain will be characterized by a given dipole displacement at each atom, which we will indicate through the vector $\vec{\xi}$. The elements ξ_i ($i = 1, .., N$ for N atoms) of this vector indicate the x (i.e. longitudinal) dipole components at each atom. By extending Eqs. 4) (main text) the splitting of this mode is:

$$\Delta\tilde{\omega} \propto \sum_{ij=1}^{N} T_{ij}^{xx} \xi_i \xi_j + O(T^2)\,. \tag{4}$$

We now Taylor expand T starting from the midpoints of the two parallel chains (which we indicate as $\bar{i}$ and $\bar{j}$, respectively), and exploit the antisymmetry of the mode, which implies $\sum_{i=1}^{N} \xi_i = 0$. After expansion one has

$$\sum_{i=1}^{N} T_{ij}^{xx} \xi_i = T_{\bar{i},\bar{j}}^{xx} \sum_{i=1}^{N} \xi_i + \partial_{x_{\bar{i}}} T_{\bar{i},\bar{j}}^{xx} \sum_{i=1}^{N} x_i \xi_i + \ldots.\,, \tag{5}$$

where x_i is the x coordinate of atom i (being $x_{\bar{i}} = 0$). Since both ξ_i and x_i are antisymmetric with respect to the midpoint of the chain, the sum is non-vanishing. By performing an analogous expansion over the other chain, one finds that the leading contribution to the exciton splitting is $\Delta\tilde{\omega} \sim \partial_{x_{\bar{i}}}\partial_{x_{\bar{j}}} T^{xx}_{\bar{i},\bar{j}} \sum_{i,j=1}^{N} x_i \xi_i x_j \xi_j$. Hence, the interchain coupling is dominated in this case by the second derivative of the dipole-dipole tensor T, that yields the correct R^{-5} scaling in the long range limit. Hence one ultimately has an effective quadrupole-quadrupole coupling.

In the case of benzene, instead, one can focus for instance on exciton 1 (see Supplementary Figure 2-**c**) in the main paper) and perform a similar Taylor expansion taking the molecular center as a reference. Due to the rotational symmetry of the mode, the summation of all linear and bilinear terms in the atomic positions (computed from the molecular center) is vanishing. As a consequence, the leading contribution arises here from $O(T^2)$ dipolar terms.

Supplementary Note 5

Density functional theory calculations were performed via the Quantum Espresso [2] simulation package, making use of norm-conserving pseudopotentials, and expanding wavefunctions in plane waves with an energy cutoff of 70 Ry. The semi-local Perdew Burke Ernzerhof [3] (PBE) exchange correlation functional was exploited. Cubic simulation cells with 35 Bohr lateral size were adopted to minimize spurious interactions with periodic replicas, and truncation of the Coulomb interaction for intrafragment direct and exchange terms was enforced in GW and BSE calculations to avoid spurious long-range exchange terms. The GLW package [4, 5], and the BSE-simple [6] code (both available within the Quantum Espresso suite) were exploited. The number of Kohn-Sham (KS) orbitals N_{bnd} adopted in the different systems is reported in Supplementary Table 1 for reference. Calculations were run at the gamma point, expanding the GW polarizability over a basis with $N_{\mathrm{w-prod}}$ auxiliary functions, that are automatically generated [5], based on the same plane-wave expansion exploited for the PBE-KS orbitals.

	N_{bnd}	$N_{\mathrm{w-prod}}$
benzene	60	500
methane	20	150
ammonia	20	150
e-butadiene	60	500
naphtalene	70	600
pyrazine	65	550
all-e-octatetraene	70	600

Supplementary Table 1: Parameters adopted for DFT/GW/BSE calculations.

A scissor, computed according to the GW gap between highest-occupied/lowest-unoccupied molecular orbitals, is introduced in the BSE-Simple calculations, where (all N_{bnd}) PBE-KS orbitals are exploited.

Concerning the MBD model parameterization, isolated-atom QDO frequencies and static polarizabilities are readily available within the MBD code for all chemical species considered in this work. However, atomic polarizabilities are rescaled in calculations proportionally to the atomic Hirshfeld volumes. The Hirshfeld volume ratios V between atoms in chemical environment and isolated atoms are taken as follows: **i)** in H monomers V is fixed to 1 given the negligible overlap; **ii)** in small molecules, namely benzene, methane, ammonia, e-butadiene, naphtalene, pyrazine and all-e-octatetraene V values were computed from first principles using the FHI-AIMS code [7], with tight basis sets and PBE functional; **iii)** in linear C chains we take V=0.87 consistently with previous work [8] on low-dimensional C nanostructures; **iv)** in the 1M5C protein, V is fixed to 0.9 for all atomic species, which provides a reasonable average estimate for large organic molecules.

Supplementary Note 6

Finally, finite temperatures may also effectively interfere with the detailed nature of the vdW interactions between excited atoms and molecules, given the coexistence of attractive and re-

pulsive states at close energetic levels. By considering the MBD Hamiltonian (Eq. 3 in the main article), and only accounting for the QDO's degrees of freedom (vibrational modes are neglected here) it is possible to derive an analytical form for the Canonical partition function Z at temperature T:

$$Z(T) = \prod_i \exp\left(-\frac{\omega_i}{2K_BT}\right)\left(1 - \exp\left(-\frac{\omega_i}{K_BT}\right)\right)^{-1} . \tag{6}$$

Here the index i runs over all collective MBD frequencies (6 in the diatomic case), and the energy expectation value is obtained as $\langle E \rangle_T = -\frac{\partial}{\partial(1/K_BT)} ln(Z)$. From Supplementary Figure 3 we observe that finite temperatures can cause an effective reduction of the interchain interaction of parallel C chains, which is however negligible at room temperature. Force reduction becomes effective only when T approaches the lowest excitation energy of the system, i.e. beyond the typical melting temperature of most materials.

In Supplementary Figure 3 we analyse the temperature dependence of interfragment energy and force between two parallel C chains containing 100 atoms each. At room temperature the occupation of excited electronic levels is negligible even in the long chain, where relatively low excitation modes are found. The impact on interfragment energy and force is only visible at the 10^4 K scale. A ground-state theory is thus sufficient for the description of interfragment forces in the absence of optical excitations. We remark that phonons were not included in the theory, and are expected to yield larger finite-temperature effects.

Supplementary Note 7

The available MBD produces exciton frequencies, after dagonalization of the MBD Hamiltonian (see Eq. 4), main paper). The code reads molecular geometry and Hirshfeld volume ratios from the input, and associates an effective polarizability and oscillator frequency to each atom. The interaction tensor is subsequently computed, and exciton frequencies are finally obtained

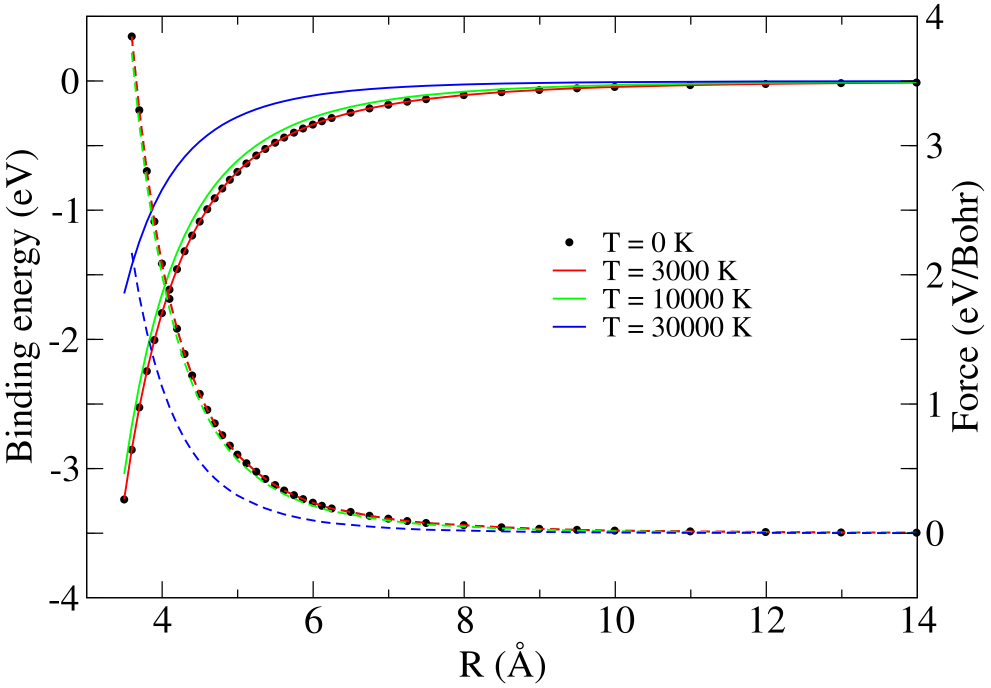

Supplementary Figure 3: Temperature dependence of the interchain dispersion energy (solid lines) and force (dashed lines) for two parallel C chains containing 100 atoms each.

by matrix diagonalization, with a computational cost that scales as $\sim N^3$.

Supplementary References

[1] Rohlfing, M., Louie, S. G. Electron-hole excitations and optical spectra from first principles. *Phys. Rev. B* **62**, 4927 (2000).

[2] P. Giannozzi, et al. Advanced capabilities for materials modelling with Quantum ESPRESSO. *J. Phys: Condens. Matter* **29**, 465901 (2017).

[3] Perdew, J.P., Burke, K., Ernzerhof, M. Generalized gradient approximation made simple. *Phys. Rev. Lett.* **77**, 3865 (1996).

[4] Marsili, M., Mosconi, E., De Angelis, F., Umari, P. Large-scale GW-BSE calculations with N^3 scaling: excitonic effects in dye-sensitized solar cells. *Phys. Rev. B* **95**, 075415 (2017).

[5] Umari, P., Stenuit, G., Baroni, S. GW quasiparticle spectra from occupied states only. *Phys. Rev. B* **81**, 115104 (2010).

[6] Prandini, G., Galante, M., Marzari, N., Umari, P. SIMPLE code: optical properties with optimal basis functions. *Comp. Phys. Comm.* **240**, 106 (2019).

[7] Blum, V. et al. Ab initio molecular simulations with numeric atom-centered orbitals. *Comput. Phys. Commun.* **180**, 2175–2196 (2009).

[8] Ambrosetti, A., Ferri, N., DiStasio Jr., R. A., Tkatchenko, A. Wavelike charge density fluctuations and van der Waals interactions at the nanoscale. *Science* **351**, 1171 (2016).
